# Living with Smoker(s) and Smoking Cessation in Chinese Adult Smokers: Cross-Sectional and Prospective Evidence from Hong Kong Population Health Survey

**DOI:** 10.3390/ijerph15010074

**Published:** 2018-01-05

**Authors:** Zhi-Ming Mai, Sai-Yin Ho, Man-Ping Wang, Lai-Ming Ho, Tai-Hing Lam

**Affiliations:** 1 School of Public Health, University of Hong Kong, Hong Kong, China; maizm@hku.hk (Z.-M.M.); syho@hku.hk (S.-Y.H.); lmho@hku.hk (L.-M.H.); 2 School of Nursing, University of Hong Kong, Hong Kong, China; mpwang@hku.hk

**Keywords:** smoking cessation, unassisted quit, living with smoker(s), adult smokers, prospective study, a Chinese general population

## Abstract

*Background*: Results on the environmental influence on unassisted quitting are scarce. We investigated the associations of living with smoker(s) with quitting in Chinese adult smokers. *Methods*: We examined both cross-sectional and prospective data in the Hong Kong Population Health Survey recruited participants in 2003/04, and followed up to 2006. Unconditional logistic regression yielded adjusted odds ratios (AORs) of (i) planning to quit, (ii) ex-smoking (cross-sectional), and quitting (prospective) for living with smoker(s). 1679 ever smokers aged 18+ years at baseline, and 323 of them who were successfully followed-up were included in the cross-sectional, and prospective analysis. *Results*: At baseline, living with smoker(s) was significantly associated with lower odds of planning to quit in current smokers (AOR 0.41, 95% CI 0.25–0.68), and lower odds of ex-smoking (AOR 0.45, 95% CI 0.34–0.58), particularly if the smoker(s) smoked inside home (AOR 0.35, 95% CI 0.26–0.47). Prospectively, living with smoker(s) non-significantly predicted lower odds of new quitting (AOR 0.48, 95% CI 0.13–1.78). *Conclusions*: Our study has provided the first evidence in a Chinese general population that living with smoker(s) is an important barrier against smoking cessation. To boost quit rate in nonusers of smoking cessation services, smoking at home should be banned, especially for populations living in crowed urban environments that are typical of economically developed cities in China.

## 1. Introduction

Tobacco smoking causes 6 million deaths each year in the world, which will continue to rise unless there is widespread cessation [1]. However, in China, where one-third of the world’s cigarettes are consumed, less than one-third of current smokers had an intention to quit [2]. In Hong Kong, the most developed and Westernized city of China, where the smoking prevalence is amongst the lowest in the world (daily smoking 10.5% in 2015) and tobacco epidemic reached its peak about 20 years earlier than in the Mainland [3], only 37% of daily smokers had an intention to quit, but most failed to quit [4].

Successful quitting is associated with many factors. Having an intention to quit is a prerequisite for preparing and taking action of smoking cessation, and it is strongly associated with successful quitting [5,6]. Several socio-demographic factors have been associated but inconsistently with quitting [7]. Smoking cessation services provided evidence-based interventions to improve quitting [8], but few smokers use these services (16.1% in Hong Kong in 2015) [4,9]. Cessation medications may be less effective in a real life general population setting than in research trials [10]. Many smokers stop smoking without any assistance, but most of the cessation studies have neglected these unassisted quitters [11]. Understanding the psychosocial and environmental influence on unassisted quitting is essential, but such evidence is scarce.

Environments that permit smoking can provide cues that prompt smoking [12]. Secondhand smoke (SHS) exposure may be a source of additional “nicotine exposure” [13], and has been associated with nicotine dependence and a lower quit rate [14,15,16,17,18]. SHS exposure may also hinder quitting through psychological effects [19]. Even though the smoking prevalence is low, SHS exposure is prevalent in nonsmokers, and also in smokers in Hong Kong [20]. Living with smoker(s) seemed to impede quitting in Chinese adult smokers who had cardiovascular illnesses [21] and healthy adolescent smokers [22], but it remains uncertain whether these associations can be generalized to the general populations, particularly the nonusers of smoking cessation services. Our literature review showed no reports on the association of living with smoker(s) with smoking cessation in a Chinese general population. We investigated the cross-sectional and prospective associations of SHS exposure and quitting in Chinese adult smokers in a real life general population setting in Hong Kong, where smoking cessation services were scarce during 2003/04 to 2006.

## 2. Materials and Methods

We examined both cross-sectional and prospective data from 7084 land-based non-institutionalized subjects in the Hong Kong Population Health Survey (PHS) that recruited participants in 2003/04 and followed up to 2006. Details about the design, methods, and subjects have been described elsewhere [23].

### 2.1. Baseline Survey

The PHS in 2003/04 aimed to collect population representative data to support health policy, using probability sampling based on the frame of quarters maintained by the Hong Kong Government Census and Statistics Department. This baseline survey recruited 3035 households with 7084 land-based non-institutionalized Chinese-speaking respondents aged 15+ years. The response rate was 72.0% at household level and 44.2% at individual level. Trained interviewers conducted face-to-face interviews using a structured questionnaire on socio-demographic status, smoking history (including SHS exposure), alcohol drinking, diet, physical activities, health knowledge and attitudes, and self-reported health status.

#### Measurements at Baseline

Planning to quit among current smokers was measured with the question “Have you seriously planned to quit in the next one month?” with responses of “Yes” and “No”. Smoking history was measured by: “Have you ever smoked cigarette?”, with responses of “I smoke daily (at least one cigarette a day)”, “I smoke occasionally (fewer than one cigarette a day)”, “I have quit already (daily previously for at least six consecutive months)”, “I used to smoke (less than one cigarette daily for six consecutive months), but have already quit” and “No, never smoked”. Those who had quit smoking at baseline were classified as “ex-smokers”, and those who smoked daily or occasionally as “current smokers”.

### 2.2. Follow-Up

**Figure 1:**
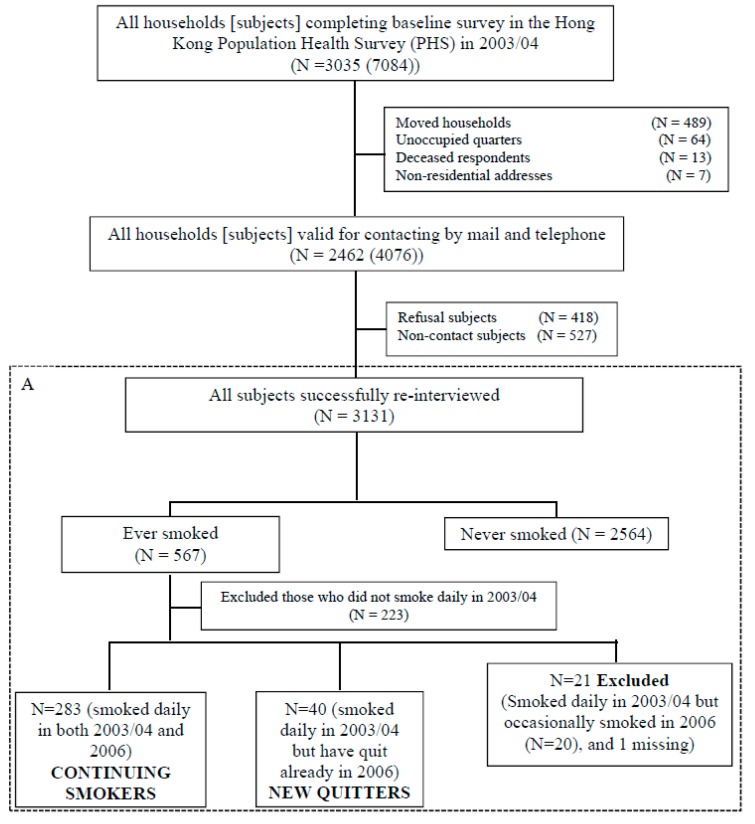
Summary of subjects included at baseline survey (2003/04), and subjects who were continuing smokers and new quitters (**A**) at follow-up.

A total of 3131 subjects (44.2% of 7084) were successfully followed in 2006 (Figure 1). The households were contacted by mail and telephone to obtain informed consent. The survey methods were the same as in 2003/04.

#### Measurements at Follow-Up

Among daily smokers at baseline, those who had quit at follow-up were classified as “new quitters”, and those who still smoked daily as “continuing smokers” (A in Figure 1). SHS exposure at home was measured at baseline survey by: “Excluding yourself, do any members of your household smoke?” with responses of “No”, “Yes, living with smoker(s) but they did not smoke inside home” and “Yes, living with smoker(s) and they smoked inside home”, and by “How many cigarettes did they smoked inside home (cigarette(s) per day)”.

### 2.3. Covariates

Socio-demographic information collected at baseline and follow-up, including sex (Female/Male), age (5-year age groups), place of birth (Hong Kong/Others), education (No formal/Primary/Secondary/Tertiary), income (No income, unemployed/No income, others (student, home-makers, retired)/<HK$10,000/10,000–19,999/≥20,000/Not willing to report; US$1 ≈ HK$7.8), and SHS exposure at workplace (No/Yes) and other places (No/Yes) was collected and treated as potential confounders.

### 2.4. Statistical Analysis

Subjects aged 18+ years at baseline were included in the analysis. Baseline cross-sectional associations of SHS exposure at home with: (i) planning to quit (“seriously planned to quit in the next 1 month” vs. “not planned” (reference group)), and (ii) ex-smoking (ex-smokers versus current smokers) were examined using unconditional logistic regression. Prospective associations of SHS exposure at home with new quitting at follow-up (new quitters versus continuing smokers) were also examined using unconditional logistic regression.

The adjusted odds ratios (AORs) were calculated based on, whether at baseline: (i) the subjects lived with smoker(s), (ii) co-residing smokers smoked inside home, and (iii) the number of cigarettes smoked by co-residing smokers inside home per day (1–5/6+ cigarettes), compared with subjects who did not live with smoker(s). All the analyses were conducted using SPSS 20.0 (IBM, New York, NY, USA).

1679 ever smokers (25.4% of 6607 subjects aged 18+) were included in the baseline cross-sectional analysis. In the prospective analysis, 323 of them at follow-up (283 continuing smokers and 40 new quitters) were included.

### 2.5. Ethics Approval and Consent to Participate

The Institutional Review Board of the University of Hong Kong/Hospital Authority Hong Kong West Cluster approved the study (UW 06-131 T/1156) and all participants gave written, informed consent before participation.

## 3. Results

Supplementary Table S1 shows that living with smoker(s) (33.1% at baseline and 30.1% at follow-up), and exposures to SHS at workplace (33.3% and 42.0%) and other places (87.0% and 92.7%) were prevalent. The cumulative quit rate up to the three-year follow-up (from 2003/04 to 2006) was 11.7% (95% CI 8.3–15.1).

Table 1 shows that at baseline, living with smoker(s) was significantly associated with lower odds of planning to quit (AOR 0.41, 95% CI 0.25–0.68, model 2). Living with smoker(s) who smoked inside home was also significantly associated with lower odds of planning to quit (AOR 0.50, 95% CI 0.30–0.82, model 2), and the AORs decreased with an increasing number of cigarettes smoked by the co-residing smokers (*p* for linear trend: 0.01 in model 1, and 0.005 in model 2).

Table 2 shows that in the ever smokers at baseline, living with smoker(s) was significantly associated with lower odds of being ex-smokers (AOR 0.45, 95% CI 0.34–0.58, model 2), particularly if the co-residing smoker(s) smoked inside home (AOR 0.35, 95% CI 0.26–0.47, model 2). Significant linear trends (*p* < 0.0001) of lower odds of being ex-smokers with increasing number of cigarettes smoked by co-residing smokers were observed.

Table 3 shows that baseline daily smokers who lived with smoker(s) were less likely, but non-significantly, to have quit smoking during follow-up (7.1%, 95% CI 4.3–9.9) than those who did not live with smoker(s) (14.7%, 95% CI 10.8–18.6). Living with smoker(s) who smoked inside home at baseline non-significantly predicted lower likelihood of new quitting (AOR 0.63, 95% CI 0.26–1.51, model 1). The AOR was reduced to 0.54 (95% CI 0.14–2.04) with further adjustments in model 2, but was also not statistically significant.

## 4. Discussion

Hong Kong has one of the lowest smoking prevalence in developed regions as a result of strong tobacco control measures. The prevalence continues to decline, but appeared to be stable at about 11% in the last five years [4]. Boosting quit rate to lower the burden of smoking-related illnesses is of public health concern, particularly in the nonusers of smoking cessation services. In our study, living with smoker(s) was significantly associated with lower odds of intention to quit and smoking cessation among ever smokers in the general population, where smoking cessation services are scarce. These associations were also observed, but became statistical non-significant in the prospective analysis, probably due to insufficient number (only seven baseline daily smokers who lived with smokers(s) had quit smoking during follow-up). Our study has provided the first evidence in a real life general population setting that living with smoker(s) is linked to lower odds of intention to quit and smoking cessation in Chinese adult smokers.

These associations were consistent with previous findings in specific populations, such as adolescents [22,24], and adults [18,21,25], particularly those with cancer [16,17]. SHS may hinder smoking cessation through physiological and psychosocial mechanisms. First, SHS exposure is biologically addictive [15]. Second, smokers who lived with other smokers and intended to quit may have more difficulties due to smokers’ etiquette, peer pressure, and camaraderie [26], and viewing smoking scenes has been linked to increased odds of smoking and relapse [27,28]. Third, smoking family members may also have a role modeling effect, and seeing family members smoke could provide cues that prompt smoking [29]. Indeed, we observed lower odds of intention to quit and smoking cessation in current smokers from living with smoker(s) only if the latter smoked inside home. However, these associations may vary in different age groups. Hong Kong was liberated in August 1945 after the “three years and eight months” under Japanese rule. Since the 1950s, the economy developed rapidly, together with rapidly increasing prevalence of smoking and cigarette consumption [4]. People born after 1945 may start smoking cigarettes younger and smoke more, and the determinants on unassisted quitting could be different. Further studies with a focus on subjects born before or after 1945 are warranted. Nicotine dependence was associated with SHS exposure [30], and smoking cessation [7]. However, information about prior nicotine dependence at baseline was not available. Such information was available in prospective analysis, but it will be difficult to examine the mediation effects of nicotine dependence on the association of living with smoker(s) with new quitting for the small number of new quitters during follow-up, though the present study is one of the largest in Chinese populations. Future studies with a larger sample size are needed to test the mediation and interaction effects of nicotine dependence.

Our results have also provided further evidence to ban smoking in public places, particularly outside workplaces, and suggested prohibiting smoking at home is beneficial to smokers for quitting smoking. Hong Kong is one of the most densely populated (6897 people/km^2^ in 2014) places in the world, where homes are typically small, making SHS exposure particularly concentrated and difficult to avoid when someone smokes. Therefore, our findings would be important for smokers who would like to quit but live in small urban homes, particularly in the most economically developed cities in China Mainland and elsewhere. Clinical doctors/health care professionals should assess SHS among smokers when delivering cessation services, and give advice on SHS avoidance.

Several limitations should be considered. First, the PHS (2003/04–2006) was conducted before the comprehensive smoke-free legislation in Hong Kong started in January 2007, which bans smoking at all indoor workplaces and most public places. Our present analysis may not be generalized to the present situation in Hong Kong. However, because smoking cessation services are still almost nonexistent in most parts of the world, the results of the present study should be relevant to show the effects of SHS exposure on quitting in many countries/regions where smoking cessation services are scarce and with less restriction on smoking. Strong support for smoking cessation is also needed to prevent the displacement of smoking into the homes when implementing comprehensive smoking ban in public places [31]. Second, our results did not differentiate the influences of SHS exposure in the users or nonusers of smoking cessation services, as such information was not collected in PHS. Smoking cessation services were scarce during 2003/04 to 2006 (only 1.6% and 2.1% of current smokers who were aware of the smoking cessation services had tried the services in 2002–03 and 2007–2008, respectively) [32,33], suggesting that the influences of the services, if any, should not be large. Third, the attrition rate was high (55.8%), which might affect the representativeness of our findings in the prospective analysis. We have found that the respondents had similar basic characteristics as non-respondents, suggesting that non-response bias should be small [23]. Few new quitters during follow-up have resulted in the wide 95% confident intervals of the risk estimates in the prospective analysis. Our prevalence of daily and ex-smoking at baseline were similar to the government statistics [32], suggesting our cross-sectional results should be representative and robust. Moreover, such problems are common in follow-up of general population samples [34]. Fourth, self-reporting bias should be considered as SHS exposure and smoking status were not biochemically verified. Validity of self-reported SHS exposure data in Hong Kong was supported by our previous studies [31]. Cotinine measures could provide objective data of SHS exposure, but they are not able to differentiate the sources of SHS exposure. Moreover, previous studies have found the effects of home, spouse, and peer SHS exposure on quitting may be substantial. Such information is not available in this study. Collecting comprehensive data on the sources of SHS exposure is warranted. Fifth, residual confounding cannot be ruled out, though some potential confounders were adjusted for. The effect size of adjusted ORs and βs for the intention to quit and smoking cessation remained similar after adjusting for place of birth, education, income, and SHS exposure at workplaces and other places.

## 5. Conclusions

This study was the first to find that living with smoker(s) is an important barrier against smoking cessation in a Chinese general population. To boost quit rate in nonusers of smoking cessation services, in addition to the users, smoking at home should be banned, especially for populations living in crowed unban environments that are typical of economically developed cities in China.

## Figures and Tables

**Table 1:** Associations of living with smoker(s) and planning to quit smoking in 1099 current smokers at baseline.

Smoking Family Members	Family Members Smoked Inside Home	Cigarettes/Day Smoked by Family Members Inside Home	Planning to Quit, *n* (%) ^a^	Model 1 AOR (95% CI) ^b^	Model 2 AOR (95% CI) ^c^
None smoked (N = 672)			125 (18.6)	Reference (1)	Reference (1)
Any smoked (N = 427)			53 (12.4)	0.55 (0.38, 0.80) **	0.41 (0.25, 0.68) **
	No		1 (2.1)	0.08 (0.01, 0.57) *	-
	Yes		52 (13.8)	0.62 (0.43, 0.90) *	0.50 (0.30, 0.82) **
		1–5	22 (5.8)	0.78 (0.47, 1.29)	0.72 (0.38, 1.35)
		6 or above	28 (7.4)	0.55 (0.35, 0.88) *	0.38 (0.19, 0.75) **
		*p* for trend		0.01 *	0.005 **

^a^ % planning to quit among current smokers. ^b^ Odds ratio of planning to quit (versus not planning to quit) adjusting for sex and age (5-year age group). ^c^ Odds ratio of planning to quit (versus not planning to quit) adjusting for sex, age (5-year age group), place of birth, education, income, and secondhand smoke exposure at work place (Yes/No) and at other places (Yes/No). AOR: adjusted odds ratios. * *p* < 0.05; ** *p* < 0.01.

**Table 2:** Associations of second-hand smoke exposure at home and between ex-smoking in 1679 ever smokers at baseline.

Smoking Family Members	Family Members Smoked Inside Home	Cigarettes/Day Smoked by Family Members Inside Home	Ex-Smokers, *n* (%) ^a^	Model 1 AOR (95% CI) ^b^	Model 2 AOR (95% CI) ^c^
None smoked (N = 1122)			441 (39.3)	Reference (1)	Reference (1)
Any smoked (N = 557)			129 (23.2)	0.47 (0.36, 0.60) ***	0.45 (0.34, 0.58) ***
	No		36 (43.4)	1.26 (0.78, 2.04)	0.96 (0.51, 1.77)
	Yes		93 (19.7)	0.38 (0.29, 0.50) ***	0.35 (0.26, 0.47) ***
		1–5	46 (9.7)	0.52 (0.36, 0.76) **	0.32 (0.18, 0.58) ***
		6 or above	43 (9.1)	0.29 (0.20, 0.42) ***	0.39 (0.23, 0.65) ***
		*p* for trend		<0.0001 ***	<0.0001 ***

^a^ % ex-smokers among ever-smokers. ^b^ Odds ratio of ex-smokers (versus current smokers) adjusting for sex and age (5-year age group). ^c^ Odds ratio of ex-smokers (versus current smokers) adjusting for sex, age (5-year age group), place of birth, education, income, and SHS exposure at work place (Yes/No) and at other places (Yes/No). ** *p* < 0.01; *** *p* < 0.001.

**Table 3:** Associations of second-hand smoke exposure at home and quitting at follow-up among 323 baseline daily smokers.

Smoking Family Members	Family Members Smoked Inside Home	Cigarettes/Day Smoked by Family Members Inside Home	Quitting, *n* (%) ^a^	Model 1 AOR (95% CI) ^b^	Model 2 AOR (95% CI) ^c^
None smoked (N = 224)			33 (14.7)	Reference (1)	Reference (1)
Any smoked (N = 99)			7 (7.1)	0.61 (0.25, 1.47)	0.48 (0.13, 1.78)
	No		0	-	-
	Yes		7 (7.4)	0.63 (0.26, 1.51)	0.54 (0.14, 2.04)
		1–5	3 (3.2)	1.03 (0.28, 3.77)	0.38 (0.04, 3.48)
		6 or above	3 (3.2)	0.38 (0.11, 1.31)	0.33 (0.04, 2.92)
		*p* for trend		0.14	0.22

^a^ % quitting at follow-up among baseline daily smokers (excluding 652 missing at follow-up). ^b^ Odds ratio of quitters (versus continuing smokers) adjusting for sex and age (5-year age group). ^c^ Odds ratio of quitters (versus continuing smokers) adjusting for sex, age (5-year age group), place of birth, education, income, and SHS exposure at work place (Yes /No) and at other places (Yes /No).

## Supplementary Materials

The following are available online at www.mdpi.com/1660-4601/15/1/74/s1, Table S1: Basic characteristics among 1679 ever smokers at baseline, and among 323 at follow-up (283 continuing and 40 ex-smokers, among 995 daily smokers at baseline excluding 20 occasional smokers and 652 non-respondents at follow-up).
