# Supplementary material for: Living with Smoker(s) and Smoking Cessation in Chinese Adult Smokers: Cross-Sectional and Prospective Evidence from Hong Kong Population Health Survey
Source: Int J Environ Res Public Health. 2018 Jan 5;15(1):74. doi: 10.3390/ijerph15010074 (PMC5800173; doi:10.3390/ijerph15010074)
Supplement: Supplementary file 1 [file ijerph-15-00074-s001.pdf]

Table S1. Basic characteristics among 1679 ever smokers at baseline, and among 323 at follow-up (283 continuing and 40 ex-smokers, among 995 daily smokers at baseline excluding 20 occasional smokers and 652 non-respondents at follow-up).

		Baseline		Follow-Up	
		n	%	*n*	%
**Mean Age (SD)**		1679	47.2 (17.0)	343	47.4 (14.1)
**Sex**	Men	1367	81.4	301	87.8
**Place of birth**	Hong Kong	916	54.6	167	48.7
	Others	763	45.4	176	51.3
**Education**	No formal	86	5.1	15	4.4
	Primary	456	27.2	104	30.3
	Secondary	915	54.5	200	58.3
	Tertiary	182	10.8	15	4.4
	Missing	39	2.3	9	2.6
**Personal income**	No income: no employment	132	7.9	36	10.5
	No income: others	494	29.4	84	24.5
	<$10,000	445	26.5	100	29.2
	$10,000-19,999	372	22.1	78	22.7
	≥$20,000	140	8.3	20	5.8
	Not willing to answer	94	5.6	24	7.0
	Missing	2	0.1	1	0.3
**Smoking**	Daily	995	59.3	283	82.5
	Occasionally	114	6.8	20	5.8
	Ex-	570	34.0	40	11.7
**Living with smoker(s)**	None	1123	66.9	239	69.7
	Yes, but they did not smoke inside home	83	4.9	4	1.2
	Yes, and they smoked inside home	471	28.1	99	28.9
	Missing	2	0.1	1	0.3
**Secondhand smoke (SHS) exposure at workplace**	None	481	28.6	76	22.2
	Exposed	559	33.3	144	42.0
	Missing	639	38.1	220	35.9
**SHS exposure at other places**	None	205	12.2	25	7.3
	Exposed	1461	87.0	318	92.7
	Missing	13	0.8	0	0
